# Amoxicillin is associated with a lower risk of further antibiotic prescriptions for lower respiratory tract infections in primary care – A database analysis spanning over 30 years

**DOI:** 10.1080/20018525.2018.1529535

**Published:** 2018-10-21

**Authors:** Marie Stolbrink, Laura J. Bonnett, John D. Blakey

**Affiliations:** a Institute of Infection and Global Health, University of Liverpool, Liverpool, UK; b Department of Biostatistics, Institute of Translational Medicine, University of Liverpool, Liverpool, UK; c Health Services Research, Institute of Psychology Health and Society, University of Liverpool, Liverpool, UK; d Royal Liverpool University Hospital, Royal Liverpool and Broadgreen University Hospital NHS Trust, Liverpool, UK

**Keywords:** Antibiotics, lower respiratory tract infections, primary care, amoxicillin, treatment failure, database

## Abstract

Antibiotic prescriptions for lower respiratory tract infections occur commonly in primary care but there is uncertainty about the most effective initial treatment strategy. Both increasing antimicrobial resistance and awareness of preventable harm from medicines make resolving this uncertainty a priority. Pragmatic, real-life epidemiological investigations are needed to inform future interventional studies.

In this cross-sectional database study we analysed antibiotic prescriptions for non-pneumonic, lower respiratory tract infections (LRTI) in primary care as captured in the Optimum Care Database from 1984 to 2017. The primary outcome was a second antibiotic prescription for a LRTI code within 14 days of index prescription, the secondary outcome further antibiotic prescription for any indication. Only individuals without chronic respiratory diseases were included. We conducted univariable analysis to identify factors associated with repeat prescriptions and generate hypotheses for forthcoming projects.

We analysed 367,188 index prescriptions for LRTI. Amoxicillin was the commonest used index drug (65.1%). In 6% a second antibiotic course coded for a further LRTI was prescribed (11.2% without this coding restriction). Further antibiotic prescriptions for LRTI were significantly associated with older age, previous smoking, seven day index courses and not using amoxicillin initially. The largest effect size was seen when amoxicillin was not used as index drug (odds ratio (OR) 1.15, *p* < 0.001). This would support current prescribing practice for amoxicillin as index drug in those without respiratory disease. Prospective studies are needed to explore the observed differences.

## Introduction

Lower respiratory tract infections (LRTI) are the commonest reason for antibiotic prescriptions in primary care across Europe and the probability of antibiotic prescription for coughs and colds by general practitioners in the United Kingdom (UK) increased by 40 % between 1999 and 2011 [1,2]. It is presumed that there are variations in prescribing practice but unclear whether these result in different patient outcomes.

Primary care is an ideal target for optimising antibiotic therapy since 74% of all UK antibiotics are prescribed here [3]. The risks of rising antimicrobial resistance and adverse effects, including infective diarrhoea, must be balanced with treatment failure [4–7].

Since real-life experiences should inform real-life decision making, we undertook this retrospective observational investigation of antibiotic prescriptions in non-pneumonic LRTI and their outcomes from 1984 to 2017. We described prescribing practice and investigated factors associated with repeat prescriptions with the aim to identify areas for further investigations.

## Methods

We carried out a cross-sectional database study drawing on retrospective, electronic medical records from the Optimum Patient Care Research Database (OPCRD). We included all subjects older than 15 years who received at least one antibiotic prescription for LRTI from 27 April 1984 to 5 January 2017. Primary outcome was a new antibiotic prescription for a LRTI READ code within 14 days of initial prescription. Secondary outcome was an antibiotic prescription for any indication within 14 days of index prescription. We excluded cases with coded diagnoses of pneumonia, chronic respiratory conditions (e.g. asthma, chronic obstructive pulmonary disease (COPD)) and those whose index antibiotic course was longer than 28 days.

The OPCRD comprised data extracted through the Optimum Patient Care (OPC) Clinical Service Evaluation (http://optimumpatientcare.org/opcrd/). The OPCRD is a research-quality primary care database with a focus on respiratory diseases. It contained anonymised, routinely recorded patient data from over 525 UK general practices. The OPCRD was approved by the Trent Multi-Centre Research Ethics Committee for clinical research use. The study protocol was approved by the OPCRD’s Anonymised Data Ethics Protocols and Transparency Committee.

Data was analysed using SPSS version 22 (IBM). Univariable analysis was carried out for gender, age, body mass index (BMI), smoking status, index antibiotic drug and index duration. Statistically significant results were defined as *p* < 0.05, 95% confidence intervals were given where appropriate. Summary statistics, odds ratios, Chi-squared and student *T*-tests were presented as appropriate for each variable based on type and distribution of data. Subgroup analysis was carried out for cases with more than one index prescription and cases from 1 January 2011 to 5 January 2017 to establish if the observed effects were maintained in current antibiotic prescribing practice.

## Results

There were 1,549,402 antibiotic prescriptions in the OPCRD from 1984 to 2017; of these 753,885 were for ‘simple’ LRTI (i.e. no underlying asthma, COPD or other chronic lung disease) in patients over 15 years. ‘Chest infection not otherwise specified’, ‘bronchitis’ and ‘lower respiratory tract infections’ were the commonest codes for LRTI (67.5%, 21.7%, 10.2% respectively). We analysed the 367,188 cases (51%) for which an index duration was clearly documented (see supplementary figure).

Of the total eligible 367,188 index prescriptions, 58.9% occurred in female patients. Mean age was 55.4 years, range 16–106 years. Mean BMI was 28.1 ± 6.1 (standard deviation, SD). 35.4% were non-smokers, 27% current smokers and 28.9% were ex-smokers (missing smoking status in 8.7%). Most prescriptions occurred from 2006 to 2010 (35.3%). Most patients received only one index prescription (59.3%), with 20.4% having two and 20.3% three or more separate index prescriptions. The commonest index duration was 7 days (74.1%), followed by 5 or 6 days (22.7%). Amoxicillin was the index antibiotic prescribed most frequently (65.1%), clarithromycin and erythromycin were the next most frequent (9.1% and 7.4% respectively). About 548 cases received 2 further antibiotic courses within 14 days of the index prescription – these were excluded from further analysis.

### Further antibiotic prescriptions for LRTI

41,227 cases had a second antibiotic prescription within 14 days of the index prescription (11.2%). More than half of the additional antibiotic prescriptions were coded for another LRTI (54.8%, 22,176 of 41,227). Most of the 22,176 s prescriptions for another LRTI code were for 7 days (75.2%). Clarithromycin, amoxicillin and doxycyline were the most commonly used second line antibiotics (23.3%, 15.5% and 15.1% respectively).

Those receiving further antibiotics for LRTI were significantly older (mean 57.5 versus 54.1 years, mean difference −3.4 (MD), 95% CI −3.6 – −3.1, *p* < 0.001) than those not receiving antibiotics (see Table 1). Current smokers had a significantly reduced risk of repeat antibiotic prescription for LRTI compared to non-smokers (OR 0.78, 95% CI 0.78–0.81, *p* < 0.001). Ex-smokers were more likely to receive repeat antibiotics than non-smokers (OR 1.04, 95% CI 1.01–1.07, *p* = 0.02). Ex-smokers were at significantly higher risk of repeat antibiotic prescription compared to current smokers (OR 1.34, 95% CI 1.29–1.39, *p* < 0.001). Not using amoxicillin as index antibiotic was associated with more repeat antibiotic prescriptions (OR 1.15, 95% CI 1.11–1.18, *p* < 0.001). Seven day index courses were associated with more repeat prescriptions than both shorter and longer courses (OR 0.93, 95% CI 0.90–0.96, *p* < 0.001; OR 0.86, 95% CI 0.79–0.94, *p* = 0.001 respectively). Higher BMI was associated with further prescriptions (mean 28.5 ± 6.3 vs 28.1 ± 6.7, MD −0.46, 95% CI −0.6 – −0.4, *p* < 0.001). There was no difference gender (*p* = 0.08).

**Table 1.** Univariable analysis for repeat antibiotic prescription with LRTI code (total number 366,640).

	Receiving second antibiotic course for LRTI			
Analysed variable	Yes	No	Calculation	*p* value
Age (years)	57.5 ± 16.6	54.1 ± 17.7	MD −3.4 (95% CI −3.6 to −3.1)	< 0.001
Smoking status				
Non-smoker	8,528 (6.6%)	121,363 (93.4%)	OR 1.00	
Current smoker	5,135 (5.2%)	93,929 (94.8%)	OR 0.78 (95% CI 0.75–0.81)	< 0.001
Ex-smoker	7,208 (6.8%)	98,737 (93.2%)	OR 1.04 (95% CI 1.01–1.07)	0.02
Missing			*n* = 31,740	
Index drug				
Amoxicillin	13,905 (5.8%)	224,716 (94.2%)	OR 1.00	
Not amoxicillin	7,323 (6.6%)	103,339 (93.4%)	OR 1.15 (95% CI 1.11–1.18)	< 0.001
Missing			*n* = 17,357	
Index antibiotic duration				
7 days	16,732 (6.2%)	254,769 (93.8%)	OR 1.00	
< 7 days	4,904 (5.8%)	80,188 (94.2%)	OR 0.93 (95% CI 0.90–0.96)	< 0.001
> 7 days	540 (5.4%)	9,507 (94.6%)	OR 0.86 (95% CI 0.79–0.94)	0.001
BMI (kg/m^2^)	28.5 ± 6.3	28.1 ± 6.7	MD −0.46 (95% CI −0.6 to −0.4)	< 0.001
Gender				
Female	12,957 (6.1%)	199,185 (93.9%)	1.00	0.08
Male	9219 (6.0%)	145,279 (94.0%)	OR 0.98 (95% CI 0.95–1.00)	

*(Mean ±SD or absolute number +row % where appropriate; SD: standard deviation; OR: odds ratio; 95% CI: 95% Confidence interval; MD: mean difference;* p *value as per students* T*-test or Chi-Square)*

**Figure 1.**
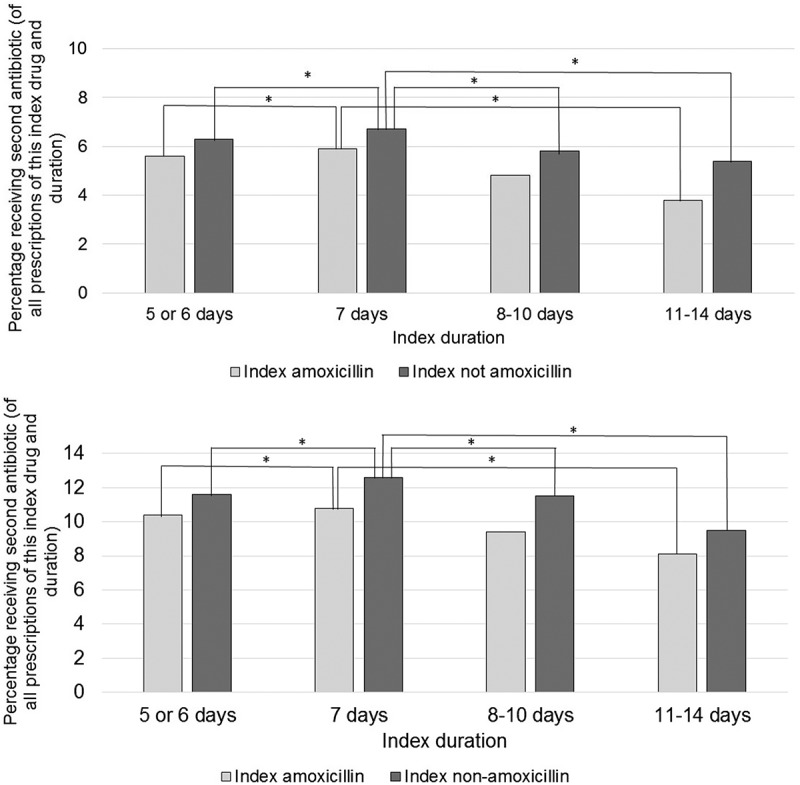
Percentage of all prescriptions of specified index duration and index antibiotic receiving second antibiotic prescription for LRTI (a) or any indication (b) within 14 days of index antibiotic prescription, by index duration and antibiotic group. (*p < 0.005 by Chi-square test).

Figure 1 shows that most repeat antibiotic prescriptions occurred following an index duration of 7 days. The proportion of cases receiving further antibiotics decreased with longer and shorter index duration. Fewer repeat prescriptions occurred when amoxicillin was used as index antibiotic.

#### Subgroup analysis for repeat LRTI code from 2011 to 2017

About 89,694 cases were included in this period. The most commonly prescribed index duration was again 7 days (83.7%).The most frequently used index antibiotics were amoxicillin (70.3%), clarithromycin (14.7%) and co-amoxiclav (4.8%).

Men were less likely to receive further antibiotics (OR 0.93, *p* = 0.01, see Supplementary Table 2). Those receiving antibiotics were significantly older (mean age 60 versus 57 years, *p* < 0.001) with a higher BMI (29.0 versus 28.6, *p* < 0.001). Current smokers were less likely to receive antibiotics (OR 0.81, 95% CI 0.76–0.87, *p* < 0.001) than non-smokers, ex-smokers more likely than non-smokers (OR 1.05, 95% CI 0.99–1.11, *p* = 0.11) and significantly more likely than current smokers (OR 1.29, 95% CI 1.20–1.38, *p* < 0.001). Not using amoxicillin as index antibiotic retained a higher risk of repeat prescription (OR 1.11, 95% CI 1.04–1.17, *p* < 0.001). In contrast to the overall study findings shorter durations were now associated with a higher risk of repeat prescription than 7 day courses (OR 1.12, 95% CI 1.05–1.20, *p* = 0.001). Longer durations maintained their lower risk of repeat antibiotics (OR 0.85, 95% CI 0.71–1.01, *p* = 0.06).

### Further antibiotic prescription for any indication

When considering all antibiotic prescriptions within 14 days of index prescription (41,227 cases) clarithromycin, amoxicillin and doxycycline were used most commonly as second line agents (20.5%, 17.0% and 13.7%).

Those receiving further courses were significantly older (mean 57.2 vs 54.0 years, *p* < 0.001) with a significantly higher BMI (mean 28.5 vs 28.1, *p* < 0.001, see Supplementary Table 3). Men were significantly less likely to receive antibiotics than women (OR 0.93, 95% CI 0.91–0.95, *p* < 0.001). Current smokers were less likely, ex-smokers more likely to receive antibiotics than non-smokers (OR 0.79, 95% CI 0.77–0.82, *p* < 0.001 and OR 1.04, 95% CI 1.01–1.07, *p* = 0.002 respectively). Ex-smokers were significantly more likely to receive further antibiotics than current smokers (OR 1.31, 95% CI 1.28–1.35, *p* < 0.001). Not using amoxicillin as index drug was associated with higher risk of repeat antibiotics (OR 1.18, 95% CI 1.16–1.21, *p* < 0.001; see Figure 1(b)). Shorter index courses were associated with fewer repeat prescriptions than 7-day index courses (OR 0.94, 95% CI 0.91–0.96, *p* < 0.001). There was no significant difference between longer and 7 day courses (OR 0.94, 95% CI 0.88–1.00, *p* = 0.06).

## Discussion

We present a report of antibiotic prescribing for LRTI in over 525 primary care practices in the United Kingdom over 33 years. More than 365,000 truly representative, index prescriptions were analysed and more than 1 in 20 received two antibiotic courses for LRTI within 14 days. Our hypothesis-generating findings support the widespread use of amoxicillin as index drug and suggest that initial courses shorter than seven days could potentially be effective, as they were associated with a lower risk of repeat prescriptions. These findings should be confirmed in interventional studies.

A key output from this study is a description of the frequency of and variation in prescribing practice for LRTI in primary care. Repeat antibiotic prescribing was common and in discrepancy to UK guidelines: the National Institute for Health and Care Excellence does not recommend routine antibiotic prescription for those presenting with likely self-limiting illnesses, including acute bronchitis, in primary care since in the majority of cases no pathogen or only respiratory viruses can be identified [8]. Yet 6.2% of our population even received two antibiotic courses for LRTI within two weeks. Treatment failure rates of 20% have been described previously but these included prescriptions for pneumonia and further antibiotics for all indications [9]. We present more specific data for LRTI, which can assist to plan future work. Most cases had only one index prescription; however, subgroup analysis of those with more index prescriptions showed no significant differences to the presented findings.

Our findings were inconclusive regarding duration, highlighting the need for interventional research. Overall, shorter and longer courses than seven days seemed to be more successful at reducing the need for further antibiotics for LRTI. Shorter courses also had a lower risk for all repeat indications. However, shorter courses were associated with increased repeat prescriptions in the subgroup analysis from 2011 to 2017, whilst longer courses maintained their superiority over seven day courses. Yet for all indications there was no difference between longer and seven day courses. With our data we are unable to establish whether this reflects changes in pathogens and disease or changes in presenting and prescribing behaviour by patients and clinicians. We did not have access to adverse effect data, which would be important for any future studies. A recent meta-analysis found an association of adverse effects and longer antibiotic duration in COPD patients [10].

Repeat prescriptions were more common in older subjects, those with a higher BMI or previous smoking history. These findings appear to be clinically relevant and plausible as, for example older patients are at higher risk of adverse outcomes from community acquired pneumonia [11]. Patients with previous smoking history are at risk of abnormal lung architecture, COPD and hence atypical bacterial infections [12,13]. Higher BMI may impair clearing of secretions but the mean difference in BMI was only 0.4 between groups which is unlikely to relate to clinical significance. Further work should be undertaken as these groups may have different needs for drugs and durations for LRTI due to co-morbidities and different lung architecture. Amoxicillin treats *Streptococcus pneumoniae*, the most common bacterial cause of LRTI, hence our finding of fewer repeat prescriptions after initial amoxicillin therapy could be clinically plausible [12]. Whilst the effect sizes were small, LRTI are very common [9]. Hence even small differences of less than 1% could theoretically result in meaningful differences in antibiotic prescriptions due to the size of the population, if they were true. It may be worth exploring this in the future.

Current smokers seemed to have a significantly reduced risk of repeat prescriptions (OR 0.78, 95% CI 0.75–0.81). Doctors may attribute cough in a current smoker to the smoking and hence not prescribe antibiotics. This is in contrast to retrospective European data where current smoking was an independent risk factor for antibiotic prescription in primary care patients presenting with cough and the increased risk of community acquired pneumonia and death of pneumococcal disease in smokers [14,15]. Yet the cough study demonstrated no improved recovery with antibiotics and subgroup analysis of a multi-centre randomised, placebo-controlled trial of amoxicillin versus placebo in LRTI showed no clinically meaningful advantage of antibiotic treatment, supporting our described UK findings [16].

We analysed electronic health records. There is a drive to use more routinely collected data, particularly in primary care research, for example from the National Institute of Health Research, and highlighted by recent successes such as the Salford Lung Study, to reduce burden on patients, bias and approximate real-world practice [17,18]. However, the reliance on primary care data is also this study’s major limitation – specifically the 49% of cases for which no index antibiotic duration was documented. The excluded cases had a lot of missing data (see Supplementary Table 4). The missing data had fewer non-smokers (8.8%) but also more missing smoking data (26%). It is unclear whether this represents a true difference in prevalence of non-smoking or failure to code an absence of disease. We were unable to comment on the repeat prescriptions in the non-included group due to too much missing data. One may suspect that documentation improved over time with advancing information technology, however, from 2011 to 2017 there were 206,455 cases in the database – and only 89,694 with a documented index duration (43%). More integrated healthcare technologies need to improve this in the future.

Other limitations include lack of information on other healthcare contacts or signs of infection and on other confounders, for example non-respiratory co-morbidities, allergies, prior antibiotic use, unattainability of total number of LRTI in the study period and inability to distinguish appropriate from inappropriate prescribing. Yet treatment decisions are often made independent of these, for example initial clinical severity scores and clinical response to therapy were not related to duration of antibiotic therapy in adults admitted with community acquired pneumonia [19]. Variations in clinical presentation did not explain the large discrepancies in antibiotic prescribing for acute cough in a cross-sectional study of 14 European countries [20]. Repeat prescriptions could have been issued due to genuine anti-microbial failure, but also due to drug failure caused by side effects or incorrect diagnoses. Better data is needed to distinguish these.

Nevertheless, we present a hypothesis-generating real-world picture of the prescribing patterns of antibiotics for LRTI over 33 years in 7% of all UK primary care practices, analysing over 365,000 cases. With evidence on effective antibiotic treatment currently lacking it is paramount to have a historical, pragmatic perspective as a basis to design safe interventional studies in the future.

## Conclusion

Antibiotics were prescribed for LRTI in people without chronic chest conditions. There was variation in practice and apparent deviation from guidelines. Amoxicillin as index drug seemed more successful in avoiding repeat antibiotic prescriptions than other agents, supporting the demonstrated current prescribing practice. The results for optimal antibiotic duration were less clear, but suggested that shorter index durations may be appropriate for LRTI. This pragmatic study has implications for further work around guideline implementation, antimicrobial resistance strategies, and interventional studies of antibiotic duration.
