# Supplementary material for: Amoxicillin is associated with a lower risk of further antibiotic prescriptions for lower respiratory tract infections in primary care – A database analysis spanning over 30 years
Source: Eur Clin Respir J. 2018 Oct 21;5(1):1529535. doi: 10.1080/20018525.2018.1529535 (PMC6201792; doi:10.1080/20018525.2018.1529535)
Supplement: Supplemental Material [file ZECR_A_1529535_SM1183.docx]

Supplementary data

Total number of antibiotic prescriptions for LRTI in OPCRD from 1984-2017

**n = 1,549,402**

Prescriptions in “simple” LRTI

**n = 753,885**

Prescriptions with clearly documented index duration

**n = 367,188**

Clearly documented index prescriptions, with maximum one repeat prescription in 14 days

**n =366,640**

Prescriptions in cases with respiratory co-morbidities or younger than 16 years

**n = 795,517**

Prescriptions without clearly documented index duration

**n = 386,697**

Second antibiotic prescription within 14 days of index prescription

**n = 41,227**

Second antibiotic prescription within 14 days of index prescription coded for LRTI

**n = 22,176**

2 repeat antibiotic prescriptions within 14 days of index prescription

**n = 548**

# Supplementary Figure 1

Flowchart of cases included from the OPCRD

# Supplementary Table 1

Supplementary Table 1: Baseline characteristics (total number 367,188)

| **Characteristic** | **N (%) or mean ± SD** |
| --- | --- |
| **Gender** |  |
| Female | 216,273 (58.9%) |
| Male | 150,915 (41.1%) |
| **Age** | 55.4 ± 18.0 |
| **BMI** | 28.1 ± 6.1 |
| **Smoking status** |  |
| Non-smoker | 130,109 (35.4%) |
| Current smoker | 99,188 (27.0%) |
| Ex-smoker | 106,210 (28.9%) |
| Missing | 31,771 (8.7%) |
| **Number of index prescriptions per patient** |  |
| 1 | 217,742 (59.3%) |
| 2 | 74,907 (20.4%) |
| ≥ 3 | 74,539 (20.3%) |
| **Year of index prescription^#^** |  |
| Earlier and up to 2000 | 60,324 (16.4%) |
| 2001-2005 | 87,532 (23.8%) |
| 2006-2010 | 129,463 (35.3%) |
| Since 2011 | 89,869 (24.5%) |
| **Index duration** |  |
| ≤ 4 days | 1,704 (0.5%) |
| 5 and 6 days | 83,477 (22.7%) |
| 7 days | 271,955 (74.1%) |
| 8-10 days | 7,188 (2.0%) |
| ≥ 11days | 2,864 (0.7%) |
| **Index antibiotic** |  |
| Amoxicillin | 238,948 (65.1%) |
| Clarithromycin | 33,307 (9.1%) |
| Erythromycin | 27,097 (7.4%) |
| Co-amoxiclav | 13,862 (3.8%) |
| Cefalexin | 12,573 (3.4%) |
| Doxycycline | 6,302 (1.7%) |
| Cefaclor | 6,274 (1.7%) |
| Ciprofloxacin | 6,087 (1.7%) |
| Oxytetracycline | 4,136 (1.1%) |
| Ampicillin | 1,218 (0.3%) |
| Missing | 17,384 (4.7%) |

(^#^one individual can be included in multiple periods)

# Supplementary Table 2

Univariable analysis for repeat LRTI code from 2011-2017 (total number = 89,694)

|  | **Receiving second antibiotic course for LRTI** | |  | |
| --- | --- | --- | --- | --- |
| **Analysed variable** | **Yes** | **No** | **Calculation** | **p value** |
| **Age** (years) | 60.3 ± 17.3 | 57.3 ± 18.5 | Mean difference: -3.0  (95 % CI -3.4 - -2.5) | <0.001 |
| **Smoking status** |  |  |  |  |
| **Non-smoker** | 3,077  (7.6 %) | 37,297  (92.4 %) | OR 1.00 |  |
| **Current smoker** | 1,301  (6.3 %) | 19,359  (93.7 %) | 0.81 (95 % CI 0.76‑0.87) | < 0.001 |
| **Ex-smoker** | 2,112  (8.0 %) | 24,410  (92.0 %) | 1.05 (95 % CI 0.99‑1.11) | 0.11 |
| Missing |  |  | 2,138  (2.4 %) |  |
| **Index drug** |  |  |  | <0.001 |
| **Amoxicillin** | 4,497  (7.1 %) | 58,538  (92.9 %) | OR 1.00 |  |
| **Not amoxicillin** | 2,009  (7.8 %) | 23,661  (92.2 %) | OR 1.11  (95 % CI 1.04-1.17) |  |
| Missing |  |  | 989  (1.1 %) |  |
| **Index antibiotic duration** |  |  |  |  |
| **7 days** | 5,433  (7.2 %) | 69,672  (92.8 %) | 1.00 |  |
| **< 7 days** | 994  (8.1 %) | 11,352  (91.9 %) | 1.12 (95 % CI 1.05‑1.20) | 0.001 |
| **> 7 days** | 139  (6.2 %) | 2,104  (93.8 %) | 0.85 (95 % CI 0.71‑1.01) | 0.06 |
| **BMI (kg/m^2^)** | 29.0 ± 6.5 | 28.6 ± 6.4 | Mean difference: -0.4  (95 % CI -0.6 - -0.3) | <0.001 |
| **Gender** |  |  |  | 0.009 |
| **Female** | 3,847  (7.5 %) | 47,330  (92.5 %) | OR 1.00 |  |
| **Male** | 2,719  (7.1 %) | 35,798  (92.9 %) | OR 0.93  (95 % CI 0.89 – 0.98) |  |

# Supplementary Table 3

Univariable analysis for repeat antibiotic prescription for any indication within 14 days (total number = 366,640)

|  | **Receiving second antibiotic course for any indication** | |  | |
| --- | --- | --- | --- | --- |
| **Analysed variable** | **Yes** | **No** | **Calculation** | **p value** |
| **Age** (years) | 57.2 ± 16.8 | 54.0 ± 17.7 | Mean difference: -3.2  (95 % CI -3.4 - -3.1) | <0.001 |
| **Smoking status** |  |  |  |  |
| **Non-smoker** | 15,418  (11.9 %) | 114,473  (88.1 %) | OR 1.00 |  |
| **Current smoker** | 9,564  (9.7 %) | 89,500  (90.3 %) | OR 0.79 (95 % CI 0.77‑0.82) | < 0.001 |
| **Ex-smoker** | 13,022  (12.3 %) | 92,923  (87.7 %) | OR 1.04 (95 % CI 1.01‑1.07) | 0.002 |
| Missing |  |  | 31,740  (8.7 %) |  |
| **Index drug** |  |  |  | <0.001 |
| **Amoxicillin** | 25,536  (10.7 %) | 213,085  (89.3 %) | OR 1.00 |  |
| **Not amoxicillin** | 13,752  (12.4 %) | 96,910  (87.6 %) | OR 1.18  (95 % CI 1.16-1.21) |  |
| Missing |  |  | 17,357  (4.7 %) |  |
| **Index antibiotic duration** |  |  |  |  |
| **7 days** | 30,977  (11.4 %) | 240,524  (88.6 %) | OR 1.00 |  |
| **< 7 days** | 9,164  (10.8 %) | 75,928  (89.2 %) | OR 0.94, (95 % CI 0.91‑0.96, | < 0.001 |
| **> 7 days** | 1,086  (10.8 %) | 8,961  (89.2 %) | OR 0.94, (95 % CI 0.88‑1.00, | 0.06 |
| **BMI (kg/m^2^)** | 28.5 ± 6.3 | 28.1 ± 6.8 | Mean difference: -0.4  (95 % CI -0.5 - -0.3) | <0.001 |
| **Gender** |  |  |  | <0.001 |
| **Female** | 24,510  (11.6 %) | 187,632  (88.4 %) | OR 1.00 |  |
| **Male** | 16,717  (10.8 %) | 137,781  (89.2 %) | OR 0.93  (95 % CI 0.91-0.95) |  |

# Supplementary table 4

Comparison of characteristics of included and excluded patients based on documentation of index antibiotic duration

| **Characteristic** | **Included patients (n=367,188)**  **N (%) or mean ± SD** | **Excluded patients**  **(n=386,697)**  **N (%) or mean ± SD** |
| --- | --- | --- |
| **Gender** |  |  |
| Female | 216,273 (58.9%) | 213,244 (59.8%) |
| Male | 150,915 (41.1%) | 173,453 (40.2%) |
| **Age** | 55.4 ± 18.0 | 56.3 ± 18.1 |
| **BMI** | 28.1 ± 6.1 | 28.1 ± 6.3 |
| **Smoking status** |  |  |
| Non-smoker | 130,109 (35.4%) | 34,029 (8.8%) |
| Current smoker | 99,188 (27.0%) | 119,489 (30.9%) |
| Ex-smoker | 106,210 (28.9%) | 132,637 (34.3%) |
| Missing | 31,771 (8.7%) | 100,542 (26.0%) |
| **Number of index prescriptions per patient** |  |  |
| 1 | 217,742 (59.3%) | 110,209 (28.5%) |
| 2 | 74,907 (20.4%) | 80,433 (20.8%) |
| ≥ 3 | 74,539 (20.3%) | 196,055 (50.7%) |
| **Year of index prescription^#^** |  |  |
| Earlier and up to 2000 | 60,324 (16.4%) | Not documented |
| 2001-2005 | 87,532 (23.8%) | Not documented |
| 2006-2010 | 129,463 (35.3%) | Not documented |
| Since 2011 | 89,869 (24.5%) | Not documented |
| **Index duration** |  |  |
| ≤ 4 days | 1,704 (0.5%) | Not documented |
| 5 and 6 days | 83,477 (22.7%) | Not documented |
| 7 days | 271,955 (74.1%) | Not documented |
| 8-10 days | 7,188 (2.0%) | Not documented |
| ≥ 11days | 2,864 (0.7%) | Not documented |
| **Index antibiotic** |  |  |
| Amoxicillin | 238,948 (65.1%) | 235,498 (60.9%) |
| Not amoxicillin | 110,308 (30.2%) | 132,250 (34.2%) |
| Missing | 17,384 (4.7%) | 18,949 (4.9%) |
| **Repeat antibiotics for LRTI** | **(n=366,640)** |  |
| Yes | 22,176 (6.0%) | Not documented |
| No | 345,012 (94.0%) | Not documented |
| **Repeat antibiotics for all indications** | **(n=366,640)** |  |
| Yes | 41,227 (11.2%) | Not documented |
| No | 325,961 (88.8%) | Not documented |
| **Repeat antibiotics for LRTI code 2011-2017** | **(n=89,694)** |  |
| Yes | 6,566 (7.3%) | Not documented |
| No | 83,128 (92.7%) | Not documented |
